# Population cellular kinetics and exposure-CSR analysis of CAR-T cells in Chinese patients with relapsed/refractory B-ALL

**DOI:** 10.3389/fphar.2026.1878984

**Published:** 2026-10-09

**Authors:** Yicong Bian, Jinjie Yuan, Chenrong Huang, Kaijie Zhang, Ling Xue, Fan Xia, Liqing Kang, Yan Wang, Hua Zhang, Lei Yu, Depei Wu, Liyan Miao

**Affiliations:** 1 Department of Pharmacy, The First Affiliated Hospital of Soochow University, Suzhou, China; 2 National Institution of Drug Clinical Trial, The First Affiliated Hospital of Soochow University, Suzhou, China; 3 Institute for Interdisciplinary Drug Research and Translational Sciences, Soochow University, Suzhou, China; 4 National Clinical Research Center for Hematologic Diseases, Jiangsu Institute of Hematology, The First Affiliated Hospital of Soochow University, Suzhou, China; 5 Shanghai Unicar-Therapy Bio-Medicine Technology Co., Ltd, Shanghai, China

**Keywords:** B-cell acute lymphoblastic leukemia, cellular kinetics, chimeric antigen receptor-T cell, cytokine-release syndrome, population pharmacokinetics

## Abstract

**Background:**

CD19-targeting chimeric antigen receptor (CAR)-T cells have demonstrated great efficacy in treating relapsed and refractory B-cell acute lymphoblastic leukemia (B-ALL). Despite the high objective response rate of CAR-T cell therapy, high-risk adverse event of cytokine-release syndrome occur unexpectedly.

**Objective:**

As living gene-modified cells, CAR-T cells exhibit unique kinetics. This study aimed to explore the relationship between CAR-T therapy-related CRS and the kinetics of CAR-T cells.

**Methods:**

In this study, data for 2184 CAR-transgene copies from 131 B-ALL patients treated with IL-6 knockdown CD19-targeting CAR-T cells were included to characterize the cellular kinetics using a nonlinear mixed-effects (NLME) model. The extrinsic and intrinsic factors that may impact the kinetics of CAR-T cells were also evaluated.

**Results:**

The CD4^+^/CD8^+^ ratio of CAR-T cell products was shown to be significantly positively correlated with T_max_, suggesting that a higher CD4^+^/CD8^+^ ratio may lead to a delay in reaching the maximum expansion, while other candidate covariates including tumor burden, CAR-T doses and comedication were not significantly associated with the kinetics of CAR-T cells. The estimated values of the cellular kinetic parameters C_max_, T_max_ and Fold_x_ were 4.696 × 10^4^ copies/μg, 13.0 days, and 127.5, respectively. The estimated AUC_0-28d_ and AUC_0-90d_ accounted for only 13.0% and 18.5% of AUC_0-inf_, respectively, indicating that there was long-term persistence of memory CAR-T cells *in vivo*. In addition, the incidence of CRS was investigated to be correlated with the early kinetics of CAR-T cells, and the optimal cutoff values of C_max_ and AUC_0-28d_ for severe CRS were 6.951 × 10^4^ copies/μg and 4.962 × 10^5^ copies/μg × days, respectively.

## 1 Introduction

Chimeric antigen receptor (CAR)-modified T Cells targeting antigen CD19 are a highly effective therapeutic approach for relapsed and refractory (r/r) B Cell acute lymphoblastic leukemia (B-ALL) (Baguet et al., 2024; Brudno et al., 2024; Shah et al., 2021; Tan et al., 2025; Zhang et al., 2023). Although the short-term objective response rate (ORR) of CAR-T cell therapy is high, the risk of post-treatment cytokine-release syndrome (CRS) remains high and unexpected. Previous studies have speculated that CRS associated with CAR-T cell therapy may be associated with CAR-T cell kinetics in patients (Baguet et al., 2024; Mueller et al., 2017; Dasyam et al., 2020), but supporting evidence remains limited. Moreover, CAR-T cell proliferation exhibits greatly interindividual variability, and how to assess CRS following CAR-T cell therapy using individual cellular kinetic parameters remains unclear.

CAR-T cells are gene-modified cells that exhibit unique cellular pharmacokinetic (PK) profiles *in vivo* (Mueller et al., 2017; Dasyam et al., 2020; Hu et al., 2025; Pan et al., 2025; Tegenge et al., 2025). Briefly, CAR-T cells are activated by specific antigens on tumor cells and undergo expansion in response to antigenic stimulation in patients (Pfeifer et al., 2022; Qi et al., 2022). After a period of expansion, CAR-T cells undergo depletion and numerical reduction, while a small subset of CAR-T cells may persist long-term *in vivo* and retain antitumor potential (Qi et al., 2022; Stein et al., 2019). As a personalized medicine, CAR-T cell proliferation is highly variable among individuals and may be influenced by multiple potential factors, including patient status, clinical treatment regimens and CAR-T cell product characteristics (Dasyam et al., 2020; Qi et al., 2022; Stein et al., 2019; Singh et al., 2021). However, how these factors affect CAR-T cellular kinetics as well as the quantitative relationship between the kinetic parameters and CRS development remain unclear.

Population pharmacokinetics (PopPK) models facilitate the characterization of drug behavior in patients and the investigation of potential factors influencing kinetics using large datasets from sparsely collected patient blood samples (Sheiner and Ludden, 1992; Dai et al., 2022; Han et al., 2024; Li et al., 2024). Given that CAR-T cell kinetics differ from those of traditional drugs, we employed a cellular kinetic model rather than a traditional compartmental model to quantify the behavior of CAR-T cells *in vivo* (Stein et al., 2019). A total of 2184 CAR-transgene copy measurements from 131 Chinese B-ALL patients treated with an IL-6 knockdown CD19-targeting CAR-T cell product were analyzed to characterize cellular kinetics using a nonlinear mixed-effects (NLME) model. The factors associated with CAR-T cell kinetics, as well as the relationship between CAR-T cell exposure and the occurrence of CRS were investigated in this study.

## 2 Methods

### 2.1 Study design and patients

Data were obtained from a single-center, one-arm, open-label study at our hospital (NCT03919240). The inclusion criteria included patients diagnosed with CD19-positive r/r B-ALL; adequate cardiac, hepatic and renal function; Karnofsky performance status greater than sixty or Eastern Cooperative Oncology Group (ECOG) performance status from zero to two; life expectancy greater than 3 months; negative pregnancy test and nonlactating status (women only). All enrolled patients received treatment with IL-6 knockdown CD19-targeting CAR-T cells, and were followed up for adverse events (AEs). Patients with sparsely collected peripheral blood (PB) samples available within 90 days post-CAR-T infusion for CAR transgene copy number quantification were included in the present retrospective analysis. This retrospective analysis was approved by the ethics committee of our hospital. All procedures were performed in accordance with the Helsinki Declaration of 1975, as revised in 2024. All participants provided written informed consent.

### 2.2 CAR-T cell products and treatment

CAR-T cells were provided by Shanghai Unicar-Therapy Bio-Medicine Technology Co., Ltd. The product were CAR-expressing T Cells consisting of an anti-CD19 single-chain antibody fragment and an IL-6-specific shRNA, with the manufacturing process conducted as previously described (Li et al., 2022; Kang et al., 2020). The quality of CAR-T cells was tested throughout the manufacturing process.

Patients enrolled in the study received an infusion of autologous IL-6 knockdown CD19-targeting CAR-T cells at a target dose of 5∼10 × 10^6^ cells/kg body weight after a preparative regimen consisting of fludarabine and cyclophosphamide (FC). The total CAR-T cell dose was administered in three sequential fractions over three consecutive days: 10% on the first day, 30% on the second day, and 60% on the third day.

### 2.3 Quantification of CAR-T cells in PB

CAR-transgene copy numbers in PB were quantified at different time points using a validated real-time quantitative polymerase chain reaction (qPCR) assay. qPCR was performed using the ABI 7500 Real-Time PCR System (Life Technologies, United States), and transgene copy numbers were calculated from a standard curve established by plasmids encoding the transgenes. The qPCR protocol was performed as described previously (Davis et al., 2021). The lower limit of quantitation (LLOQ) of the assay was 10 copies/μg DNA.

### 2.4 Kinetics modeling of CAR-T cells

#### 2.4.1 Base model

The base structural model was adapted from a previously published cellular kinetics model (Stein et al., 2019). All kinetics model analyses were performed using Phoenix NLME software version 8.2 (Pharsight, Certara Inc.). The typical CAR-T cellular kinetics were simplified into three processes: exponential expansion, rapid initial decline, and slow long-term decline. A schematic representation of the cellular kinetics of CAR-T cells *in vivo* is shown in Figure 1. Six key cellular kinetic parameters, namely, C_max_, T_max_, α, β, Fold_x_, and F_B_ *,* were estimated with this model. Specifically, the structural model defined ρ as the exponential expansion rate constant, which applies until the time point (T_ma*x*_) when the transgene copy number reaches its maximum value (C_ma*x*_). After T_ma*x*_, a biexponential decline was modeled, characterized by two rate constants: α (contraction) and β (persistence). To quantify the total transgene copy number over time following CAR-T cell infusion, three parameters (*R*_*0*_ *, A, B*) were incorporated into Equation 1. R_*0*_ represents the baseline transgene copy number level; A and B represent the portion of C_ma*x*_ that decline rapidly (at slope α) and gradually (at slope β), respectively, at time T_ma*x*_. Mathematical expressions for these parameters are provided in Equations 2–4. The fold expansion of CAR-T cells relative to baseline (Fold_*x*_) is described in Equation 5. The fraction of transgene copies that declined at the rate of *β* at T_ma*x*_ (F_*B*_) and the fraction of transgene copies declining at the rate of *α* at T_ma*x*_ (1 − F_*B*_) are described in Equation 6.

$$F\left(t\right)=\left\{\begin{array}{c} R_{0}\cdot e^{\rho \cdot t},t<T_{\max}\\ {A\cdot e}^{-\alpha \;\left(t-T_{\max}\right)}+{B\cdot e}^{-\beta \;\cdot \left(t-T_{\max}\right)},t\geq T_{\max} \end{array}\right.$$ (1)

$$R_{0}=C_{\max}/e^{\rho \cdot T_{\max}}$$ (2)

$$A=C_{\max}\cdot \left(1-F_{B}\right)$$ (3)

$$B=C_{\max}\cdot F_{B}\;$$ (4)

$${\mathrm{Fold}}_{x}=e^{\rho \cdot T_{\max}}$$ (5)

FB=BCmax (6)

$$Eobs=E\cdot e^{EEps}$$ (7)

**FIGURE 1:**
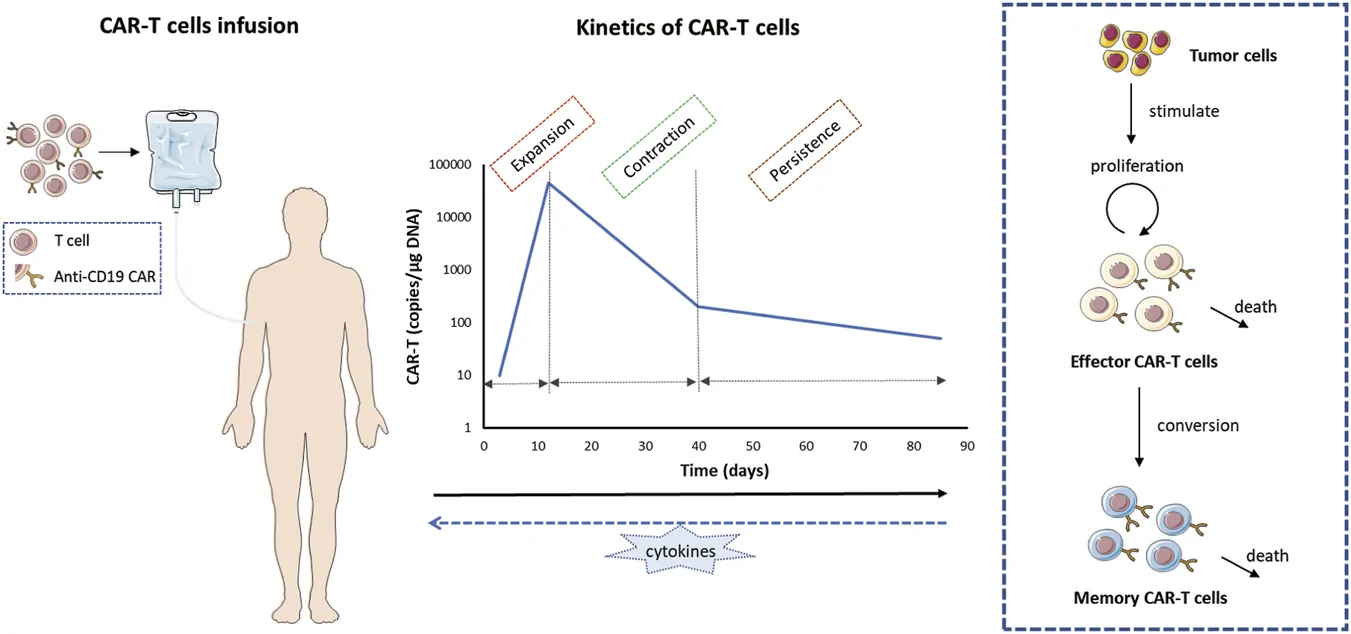
A schematic representation of the cellular kinetics of CAR-T cells *in vivo*.

Typical cellular kinetic parameters and their random interindividual variability (IIV) were estimated using a first-order conditional estimation method with the extended least squares method (FOCE ELS). Random effects were estimated via an exponential model. For the residual error model (Equation 7), natural logarithmic-transformed data were modeled with an additive error model in consideration of the magnitude difference in transgene copies.

#### 2.4.2 Covariate screening

To evaluate the distribution of covariates in the population, exploratory covariate analysis was conducted based on clinical plausibility and scatter plots of covariates versus random-effects estimates (Ette and Ludden; Wu et al., 2019). Covariates were investigated using a stepwise procedure in the model, including: demographic characteristics (sex, age, height, weight, BMI); pre-infusion tumor burden (primitive naive cells); CAR-T doses and comedication (corticosteroids or tocilizumab); *in vitro* CAR-T cell proliferative properties (culture time, proliferation fold change, cell cycle), and transduction efficiency and CD4^+^/CD8^+^ T Cell ratio of CAR-T cell products. Categorical covariates were coded as numbers, and continuous covariates were centered at their median value (Ma et al., 2020). During forward selection, a decrease in the objective function value (OFV) exceeding 3.84 (*p* < 0.05) served as the covariate inclusion criterion. For backward elimination, an OFV increase greater than 6.635 (*p* < 0.01) was defined as covariate retention criterion.

#### 2.4.3 Model evaluation

Model evaluation was based on the Goodness-of-fit (GOF) plots of the final model using the Phoenix NLME, including conditional weighted residuals (CWRES) over population predicted concentration (PRED) or time after dosing (TAD) and the relationship between observed concentration (OBS) and PRED or individual predicted concentration (IPRED) (Dai et al., 2022; Wu et al., 2019; Hooker et al., 2007; Karlsson and Savic, 2007). Visual predictive check (VPC) and bootstrap analysis (1000 runs) were also performed. Normalized prediction distribution error (NPDE) plots were generated using R software (version 4.6.1).

### 2.5 Safety assessment

Vital signs and clinical laboratory tests were included in the safety assessment. Special attention was paid to the incidence of CRS in this study. The AEs were graded according to the National Cancer Institute CTCAE version 4.03, with grade 3 or higher considered serious.

### 2.6 Statistical analysis

Patient characteristics are presented using descriptive statistics, and nonparametric tests and Fisher’s exact tests were used for comparisons among groups. The PK parameters C_max_, T_max_, α, β, Fold_x_ and F_B_ were estimated in the cellular kinetic model described above, while the area under the concentration-time curve (AUC) from time zero to day 28 or day 90 post-dose (AUC_0-28day_, AUC_0-90day_) and the AUC form time zero to infinity (*AUC*_*0-*_ ∞ ) were calculated from the above parameters (Stein et al., 2019).

The correlation between the CRS of CAR-T cell therapy and cellular kinetics was assessed using the nonparametric Mann-Whitney test. P < 0.05 were considered statistically significant. The optimal cutoff points of kinetics parameters for predicting the toxicity of CAR-T cell therapy were determined using receiver operating characteristic (ROC) curve analyses, and 95% confidence intervals (CIs) were calculated for all ROC AUCs. Data were analyzed using GraphPad Prism 9.3 software.

## 3 Results

### 3.1 Patients and CAR-T cell products

A total of 131 B-ALL patients who received IL-6 knockdown CD19-targeting CAR-T cell therapy and had quantifiable CAR-transgene copies in PB within 90 days after CAR-T cell treatment were included in the study. All patients received a dose of 5 × 10^6^∼10 × 10^6^ cells/kg CAR-T cells. The characteristics of the patients and CAR-T cell products are shown in Table 1.

**TABLE 1:** Characteristics of 131 Chinese r/r B-ALL patients and CAR-T cell products.

Characteristics	N (%)/Median (IQR, min ∼ max)
Patients	
Male (N)	72 (54.96)
Age (years)	36 (24.44, 11∼68)
Height (cm)	167 (10.5, 150∼190)
Weight (kg)	61 (17.85, 22∼110)
BMI (kg/m^2^)	21.72 (5.22, 8.59∼34.44)
Tumor burden (%)	1.00 (3.50, 0.00∼90.50)
Lymphodepleting chemotherapy with FC (%)	131 (100%)
Weight-adjusted dose of CAR-T cells (cells/kg)	5.00 × 10^6^ (0, 5.00 × 10^6^∼10.00 × 10^6^)
Total dose of CAR-T cells (cells)	3.25 × 10^8^ (1.34 × 10^8^, 1.10 × 10^8^∼9.60 × 10^8^)
Received steroids (N)	10 (7.63)
Received tocilizumab (N)	2 (1.53)
CAR-T cell products	
Transduction efficiency (%)	44.60 (22.99, 5.80∼77.12)
CD4^+^/CD8^+^ T Cell ratio	1.95 (3.91, 0.05∼25.17)
Culture time *in vitro* (days)	11 (4, 7∼20)
Proliferation fold change *in vitro*	40.19 (47.87, 6.28∼357.50)
Cell cycle *in vitro* (days)	2.10 (0.76, 1.27∼5.05)

### 3.2 CAR-T cellular kinetics

A total of 2184 observed CAR-transgene copies from 131 patients were sparsely collected within 90 days after CAR-T cells infusion. Each patient had an average of 16–17 observations, with 88.8% of the total observations obtained from the first day to the 28th day after CAR-T cells infusion.

In the preliminary estimates of the base model, the value of β was fixed at 0.001 due to high variable coefficient (43.96%). The shrinkage values of C_max_, T_max_ and Fold_x_ were 0.147, 0.240 and 0.175, respectively, indicating that the dataset used for model development was enough to compute theses parameters. In addition, the shrinkage values of *α* and F_B_ were larger than 0.5, and that of *β* was close to 1. Therefore, random effects of *α, β* and F_B_ were not incorporated into the base model. Through rigorous screening, the CD4^+^/CD8^+^ T Cell ratio in the CAR-T cell products was shown to be positively correlated to the parameter of T_max_, while other candidate covariates, including tumor burden, CAR-T doses and comedication were not significantly associated with the kinetic parameters (Figure 2; Supplementary Figure S1). Therefore, the CD4^+^/CD8^+^ T Cell ratio was included in the final model (Δ-2LL = 9, *p* < 0.01). The predictive performance of the model in terms of the GOF and VPC is shown in Figure 3, which demonstrated the exactitude of the parameter estimates and the reliability of the final model. The mean (−0.014), variance (1.04), skewness (0.088), and kurtosis (0.21) of the NPDEs collectively suggested their distribution closely matched the standard normal distribution. Accordingly, the final model exhibited no systematic bias, with no overall overprediction or underprediction of observed cellular concentrations. The final model also characterized the data well in individuals, and the typical fitting plots are shown in Figure 4.

**FIGURE 2:**
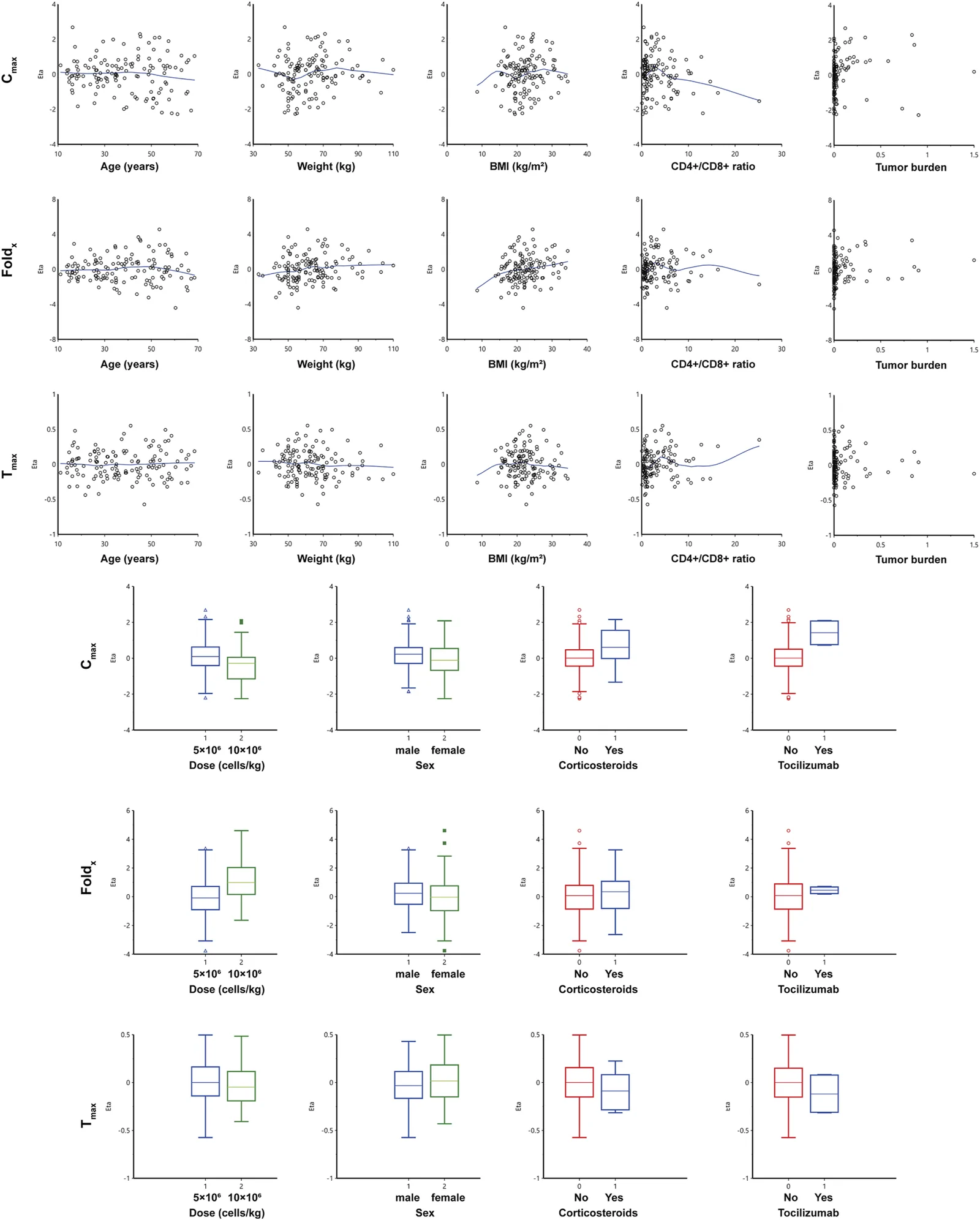
Exploratory analysis of continuous covariates and categorical covariates plotted against *post hoc* random-effects estimates.

**FIGURE 3:**
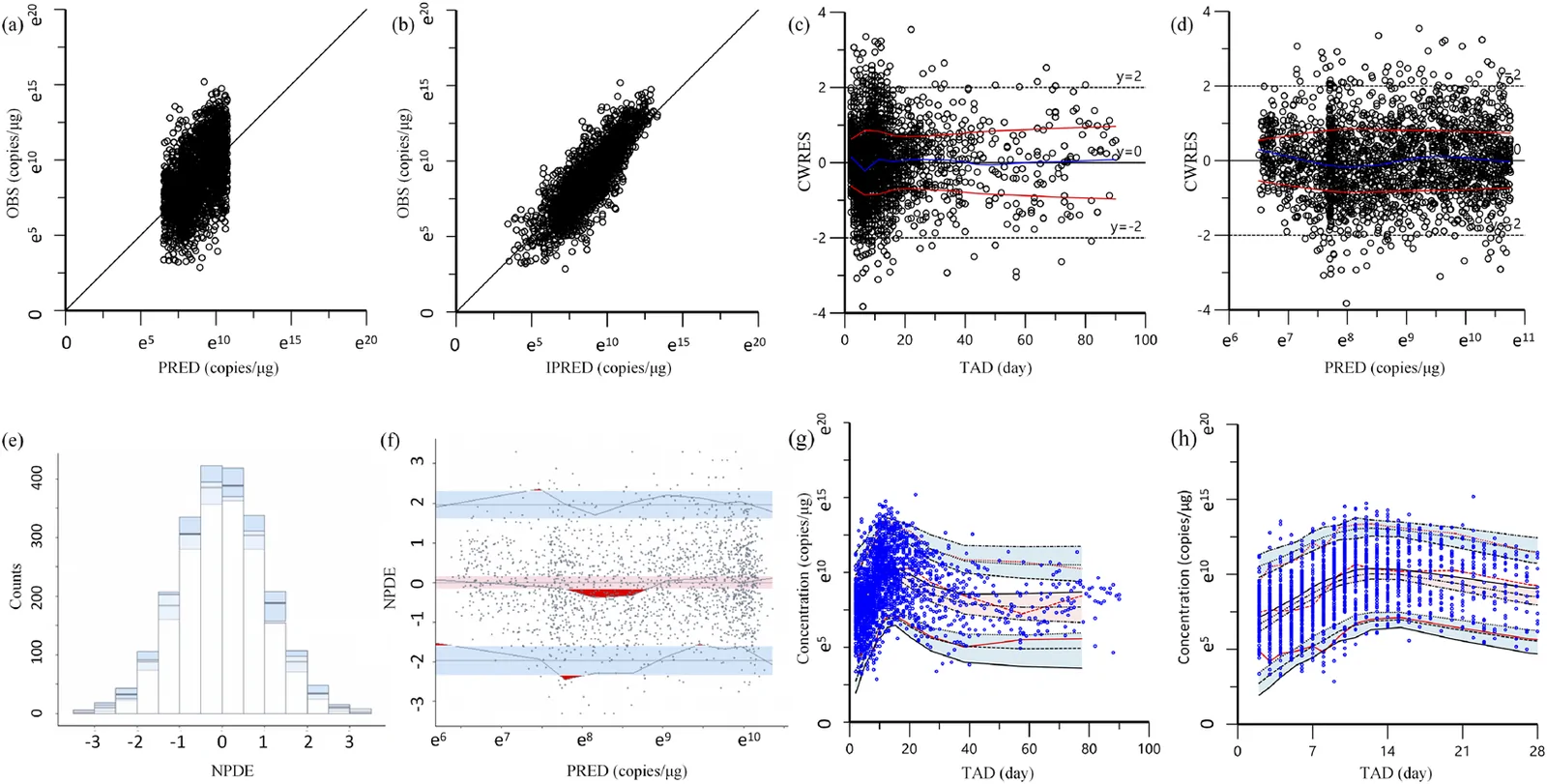
Goodness-of-fit plots (GOF) and visual prediction check (VPC) of the final model. **(a)** OBS vs. PRED and **(b)** OBS vs. IPRED. OBS, observed values. PRED, population predicted values. IPRED, individual predicted values. The points were uniformly distributed around the line y = x. **(c)** CWRES vs. TAD and **(d)** CWRES vs. PRED. CWRES, conditional weighted residuals. TAD, time after dose. The weighted residuals were mainly concentrated on both sides of y = 0, within the range of ±4. **(e)** Histogram of NPDE and **(f)** NPDE vs. PRED. NPDE, normalized prediction distribution error. The distribution of the NPDEs approximated a normal distribution, and the NPDEs were randomly and symmetrically scattered around the line y = 0 across the entire predicted concentration range, showing no discernible trend or systematic bias **(g,h)** visual prediction check within 90 days and 28 days of the final model. The blue dots denoted the observed values. The red line represented the 5th, 50th and 95th percentiles of the observed concentrations. The black lines represented 5th, 50th and 95th percentiles of simulated concentrations. The shaded areas represented 95% confidence interval of the 5th, 50th and 95th prediction intervals. The 5th, 50th and 95th percentiles and these 95% CI of the observations remain within the corresponding percentile range and predicted intervals of the predicted data from the final model.

**FIGURE 4:**
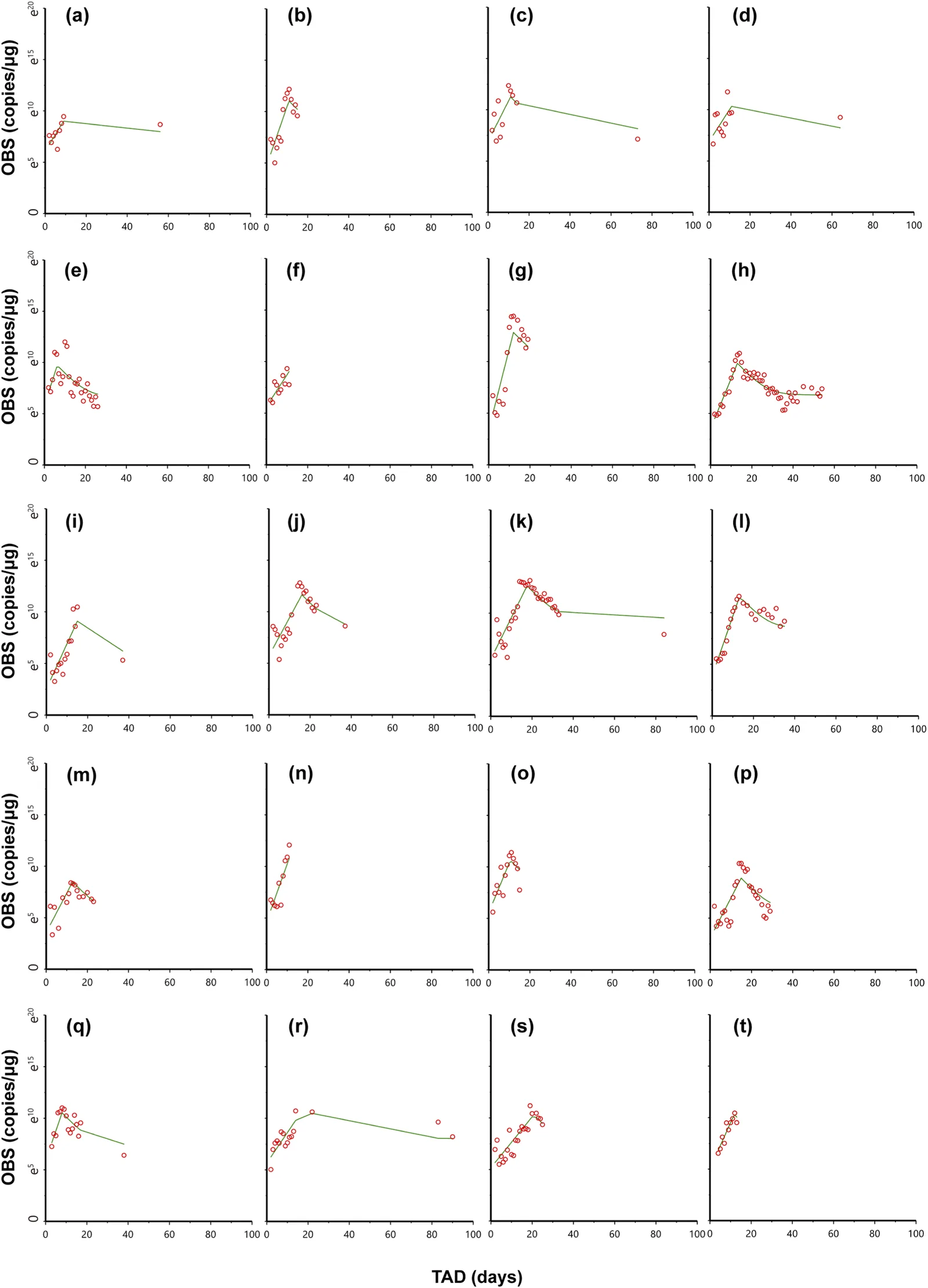
Individual fitting plots for OBS/IPRED vs. TAD. **(a–t)** represent individual subjects. OBS, observed values, IPRED, individual predicted values, TAD, time after dose. The red dots denoted the observed values, and the green line denotes the individual predicted values.

Population cellular kinetic parameters and estimation precision of both the base model and final model are presented in Table 2. The estimated cellular kinetic parameters of C_max_, T_max_ and Fold_x_ in the final model were 4.696 × 10^4^ copies/μg, 13.0 days and 127.5, respectively. The expansion rate constant *ρ* and the half-life of the rapid contraction rate (ln2/*α*) were calculated as 0.374/day and 3.077 days, while the half-life of the gradual decrease rate (ln2/*β*) could not be estimated due to the fixed value of *β*. As shown in Table 2. The parameters of *AUC*_*0-*_ ∞ of CAR-T cells was estimated as 4.610 × 10^6^ copies/μg × days on average, with a range from 2.805 ×10^5^ to 3.924 ×10^7^ copies/μg × days, and the estimated AUC_0-28day_ and AUC_0-90day_ accounted for only 13.0% and 18.5% of the *AUC*_*0-*_ ∞ , respectively.

**TABLE 2:** Population cellular kinetic parameter estimates and bootstrap results.

​	Base model				Final model				1000 bootstrap replicates			
​	Estimate	CV%	2.5% CI	97.5% CI	Estimate	CV%	2.5% CI	97.5% CI	Median	CV%	2.5% CI	97.5% CI
Kinetic parameters												
C_max_ (copies/μg)	4.519 × 10^4^	13.008	3.366 × 10^4^	5.672 × 10^4^	4.696 × 10^4^	14.441	3.366 × 10^4^	6.025 × 10^4^	4.571 × 10^4^	13.993	3.474 × 10^4^	6.148 × 10^4^
T_max_ (days)	12.400	2.293	11.842	12.957	12.950	2.767	12.247	13.652	12.515	3.227	11.705	13.337
Fold_x_	133.117	17.774	86.715	179.519	127.480	19.878	77.784	177.176	123.517	24.489	71.596	197.024
Alpha (1/day)	0.207	5.165	0.186	0.228	0.225	8.087	0.190	0.261	0.213	13.429	0.159	0.272
Beta (1/day)	0.001	/	/	/	0.001	/	0.001	0.001	0.001	/	0.001	0.001
FB	0.051	7.710	0.043	0.058	0.050	8.144	0.042	0.058	0.049	27.606	0.027	0.080
Covariate effect on T_max_												
CD4^+^/CD8^+^ T Cell ratio	/	/	/	/	0.092	23.546	0.049	0.134	0.090	23.161	0.037	0.110
Inter-individual variability												
*ωC*_*max*_	1.172	20.866	0.693	1.651	1.152	20.564	0.688	1.617	1.325	18.032	0.744	1.558
*ωT*_*max*_	0.261	3.873	0.241	0.281	0.292	7.006	0.252	0.332	0.071	4.912	0.242	0.293
ωFold_x_	1.843	36.675	0.518	3.167	1.808	36.484	0.515	3.101	3.325	37.265	0.492	3.155
Random residual variability												
Epsilon (*ε*)	1.298	1.098	1.270	1.326	1.295	1.123	1.266	1.323	1.300	2.925	1.223	1.376

### 3.3 Correlation between CRS and CAR-T cell exposure

Among the 131 patients, 72 patients (55.0%) developed CRS. Mild to moderate CRS (grade 1-2) occurred in 63 patients (48.1%), and severe CRS (grade 3-4) occurred in nine patients (6.9%). Baseline characteristics were comparable between patients with and without severe CRS. The severe CRS group consisted of four males and five females (mean age, 41 years; mean body weight, 60 kg), while patients without severe CRS included 68 males and 54 females (mean age, 37 years; mean body weight, 62 kg). The cellular kinetic parameters were analyzed between patients with and without severe CRS, and the results showed that patients who developed severe CRS had significantly higher C_max_, AUC_0-28day_ and *AUC*_*0-*_ ∞ after CAR-T cell therapy (P = 0.0004, 0.0007 and 0.0004, respectively), as shown in Figure 5. These results indicated that high exposure to CAR-T cells *in vivo* was associated with a higher risk of CRS incidence.

**FIGURE 5:**
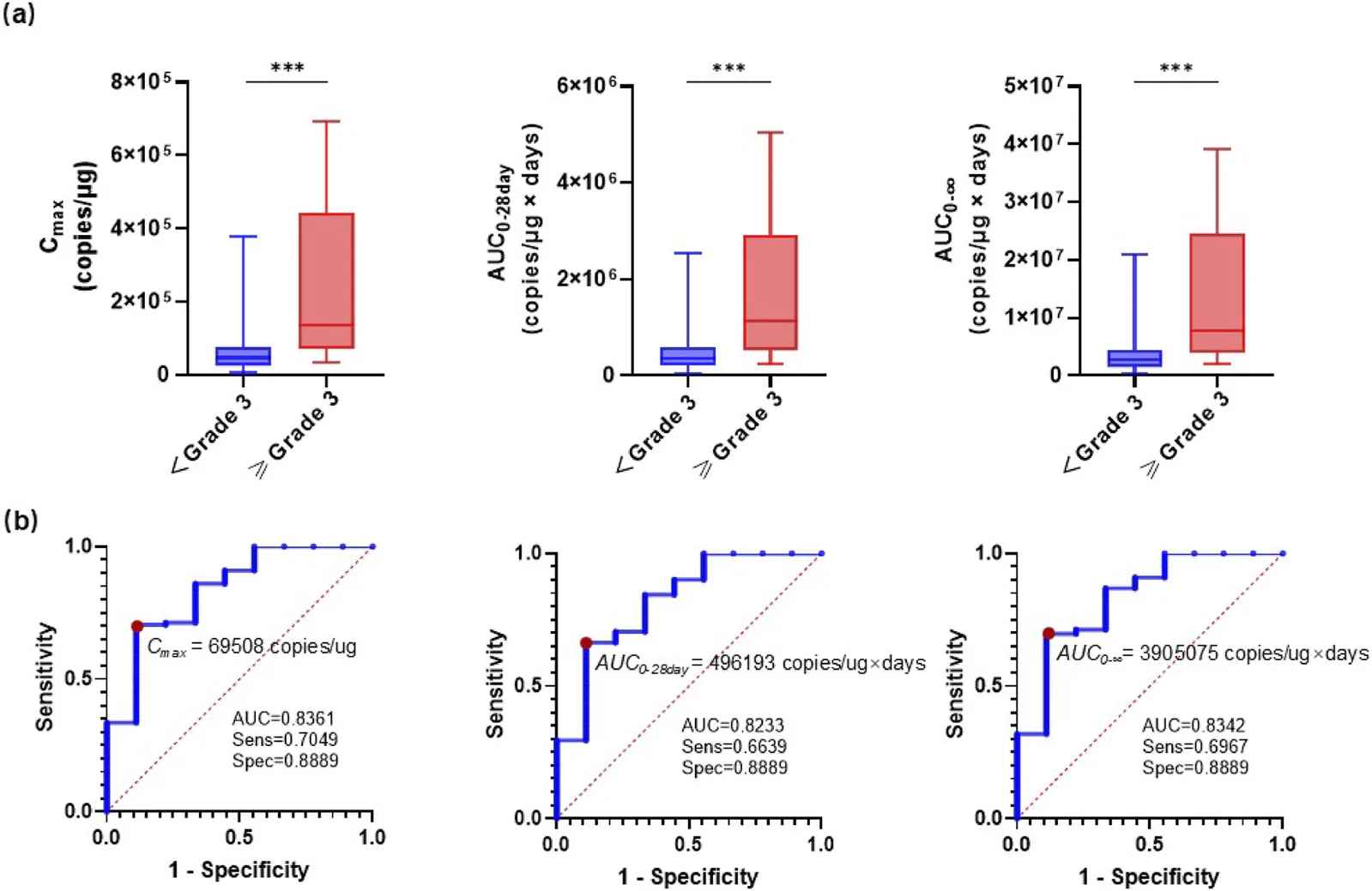
Correlation of CAR-T cellular kinetics with the incidence of CRS **(a)** C_max_, AUC_0-28day_ and *AUC*_*0-*_ ∞ of CAR-T cells in patients with moderate (n = 63) and severe (n = 9) CRS; **(b)** ROC curve analysis of C_max_ vs. CRS, AUC_0-28day_ vs. CRS and *AUC*_*0-*_ ∞ vs. CRS.

ROC curves were constructed to determine the optimal cutoff points of C_max_, AUC_0-28day_ and *AUC*_*0-*_ ∞ for severe CRS in CAR-T cell therapy. As presented in Figure 5, the areas under the ROC curves for C_max_, AUC_0-28day_ and *AUC*_*0-*_ ∞ of CAR-T cells were 0.8361 (95% CI 0.6947–0.9775, *p* = 0.0008), 0.8233 (95% CI 0.6730–0.9736, *p* = 0.0012) and 0.8342 (95% CI 0.6897–0.9788, *p* = 0.0008), respectively, and the cutoff values were 6.951 × 10^4^ copies/μg, 4.962 × 10^5^ copies/μg × days and 3.905 × 10^6^ copies/μg × days. The specificities of the cutoff values for C_max_, AUC_0-28day_ and *AUC*_*0-*_ ∞ were all 0.8889, and the corresponding sensitivities for C_max_, AUC_0-28day_ and *AUC*_*0-*_ ∞ were 0.7049, 0.6639 and 0.6967, respectively. These results suggested that exposure to CAR-T cells *in vivo* could be used to effectively evaluate the occurrence of CRS.

## 4 Discussion

In this study, we developed a population cellular kinetic model to characterize the *in vivo* kinetics of an IL-6 knockdown CD19-targeted CAR-T cell product in Chinese patients with B-ALL. Notably, we report the first systematic population cellular kinetic characterization of this unique CAR-T construct administered via a three-day split-infusion regimen in a Chinese patient cohort. As reflected in the model, the CAR-T cellular kinetics process was divided into three stages, including exponential expansion, rapid contraction and long-term persistence, which fitted perfectly with the dataset in this study and was consistent with previous study (Stein et al., 2019). Although CAR-T cells have been reported to exhibit a transient decline due to tissue distribution after the first infusion (Hardiansy et al., 2019), the early distribution phase has only been observed in a limited number of studies (Liu et al., 2021). In the current work, no obvious early distribution phase of CAR-T cells was detected, which may be partly due to the continuous infusion of CAR-T cells masking the early distribution. Thus, we selected the cellular kinetic model without the distribution phase.

Samples collected over 90 days captured the phases of exponential expansion, rapid contraction and the early stage of long-term decline. Most sampling timepoints fell within 30 days post-infusion, providing sufficient data to characterize CAR-T expansion and subsequent decline kinetics. Key parameters assessed included C_max_, T_max_ (with covariate analysis) and Fold_x_. The shrinkage values of *α*, *β*, and FB were high and similar to those reported by Stein et al. (Stein et al., 2019). High shrinkage indicates that individual empirical Bayes estimates regress strongly toward population typical values. Thus, reliable quantification of inter-individual variability for these parameters were challenging, and investigating covariate effects on these parameters was not recommended. In the base model, *β* was initially estimated at 0.001 (CV 43.96%), but its inter-individual variance could not be stably estimated across multiple initial value trials (shrinkage >0.5). Given that only approximately 10% of samples were obtained after day 30 with sparse long-term elimination phase data, β was fixed at 0.001 to guarantee model convergence and avoid over-parameterization. This value also matches the magnitude reported by Stein et al. (Stein et al., 2019).

Key parameters of C_max_, T_max_ and AUC_0-28day_ are commonly employed to characterize the early PK profiles of CAR-T cells. Our results demonstrate that CAR-T cells expand in patients and reach a peak at approximately 13 days, with the peak CAR-T cell count reaching 127.5-fold of the baseline. The estimated parameters of C_max_ and T_max_ were comparable to those in prior studies, while the value of Fold_x_ was lower (Stein et al., 2019; Ogasawara et al., 2021). The difference in Fold_x_ may be attributed to the implementation of a three-day continuous CAR-T cell infusion protocol in this study. Alternatively, it may also stem from differences in CAR-T cell products and patient ethnicity between the two studies. The estimated AUC_0-28day_ and AUC_0-90day_ accounted for only 13.0% and 18.5% of *AUC*_*0-*_ ∞ , respectively, indicating that there was long-term persistence of memory CAR-T cells *in vivo*, which was consistent with previous studies (Mueller et al., 2017).

A range of factors may affect CAR-T cellular kinetics, including but not limited to CAR design, patient disease type, tumor burden, lymphodepleting chemotherapy and concomitant medication (Dasyam et al., 2020; Qi et al., 2022; Stein et al., 2019; Singh et al., 2021). In this study, all patients enrolled were diagnosed with B-ALL and underwent FC lymphodepleting conditioning, followed by infusion of CAR-T cells manufactured via a standardized process and supplied by the same company, which means that CAR design, disease type and conditioning regimens of patients were relatively unitary in the study. Following rigorous covariate screening, CD4^+^/CD8^+^ T Cell ratio of the administered CAR-T cell products was found to exert a significant effect on T_max_, while patient characteristics, clinical treatment with steroids or tocilizumab, and other characteristics of CAR-T cell products were not significantly associated with cellular kinetics.

Unlike traditional drugs, the dose-exposure relationship of CAR-T cells was not observed in our study, as evidenced by the lack of correlation between dose and C_max_. The weak dose-exposure relationship might be explained by the proliferative capacity of CAR-T cells, and is consistent with findings from previous studies (Qi et al., 2022; Sheih et al., 2020). Furthermore, no significant correlation between tumor burden and cell expansion was observed, and cotreatment with tocilizumab or corticosteroids had no impact on CAR-T cellular kinetics, which was consistent with some published literature (Stein et al., 2019; Li et al., 2022; Liu et al., 2021; Porter et al., 2015; C et al., 2019), but not with others (Hardiansy et al., 2019; Mu et al., 2022). The impacts of CAR-T cell product characteristics on cellular kinetics were also investigated. Our results showed that a higher CD4^+^/CD8^+^ T Cell ratio of the CAR-T cell product may delay the achievement of maximum CAR-T cell expansion. Additionally, the relationship between the CD4^+^/CD8^+^ T Cell ratios of CAR-T cell products and C_max_ was presented as a polyline with a zenith, suggesting that a balanced proportion of CD4^+^ and CD8^+^ CAR-T cells could promote *in vivo* proliferation. The mechanism underlying the observation remains unclear. We propose that a favorable CD4^+^/CD8^+^ T Cell ratio coordinates the function of helper and cytotoxic CAR-T subsets. CD4^+^ CAR-T cells constantly produce supportive cytokines that sustain persistent CAR-T cell proliferation and attenuate early cellular contraction, which may ultimately delay the T_max_ of CAR-T cells in peripheral blood (Liu et al., 2026; Lee et al., 2023; Boulch et al., 2023; Bove et al., 2024). It should be noted that limited statistical power may hinder the identification of covariates with modest yet clinically meaningful effects. Larger cohorts are required to validate these potential associations in future investigations.

Furthermore, we exploratively identified C_max_ and AUC_0-28day_ cutoff values associated with the occurrence of severe CRS, providing quantitative evidence to guide clinical safety management of this novel CAR-T cell product. Generally, CRS and immune effector cell-associated neurotoxicity syndrome (ICANS) are recognized as the most important CAR-T cell related toxicities. While only two patients developed mild ICANS (grade 1) in our study, CRS was analyzed as the only toxicity index in our study. C_max_ and AUC_0-28day_ are the most commonly used pharmacokinetic parameters to characterize the early exposure of CAR-T cells. Our results suggested that high exposure of CAR-T cells *in vivo* was associated with a high risk of severe CRS and that patients with C_max_ and AUC_0-28day_ above the cutoff values of 6.951 × 10^4^ copies/μg and 4.962 × 10^5^ copies/μg × days tended to develop severe CRS due to CAR-T cell therapy. These two kinetic parameters may function as risk-stratification biomarkers in clinical practice. Patients with values above the thresholds may face a markedly higher risk of severe CRS, requiring intensified clinical monitoring and prompt implementation of standardized early intervention protocols. However, it should be emphasized that the present study only identifies an association between exposure and toxicity and cannot establish causality. Given that CRS is driven by robust T Cell activation and the systemic release of IL-6-dominated cytokines (Rial Saborido et al., 2022; Jiang et al., 2021; Jia et al., 2021), the specificity of these cutoff values may be validated in other CAR-T products, as the CAR-T cells in this study featured targeted IL-6 knockdown.

We further assessed correlations between CAR-T cell therapy response and early cellular expansion. The responders trended toward higher C_max_ and AUC_0-28day_ than nonresponders, yet no clear link emerged between 12-month clinical response and CAR-T cellular kinetics (data not shown). The pronounced interindividual variability in kinetic parameters precludes definitive conclusions on the kinetics-efficacy relationship, and longer-term follow-up is ongoing to elucidate this association. Given the high interindividual variability in CAR-T cellular kinetics, it remains challenging to draw definitive conclusions regarding the relationship between CAR-T cellular kinetics and clinical efficacy.

The main limitations are outlined below. First, this single-center retrospective cohort exclusively enrolled Chinese patients, and its generalizability is restricted by the homogeneous ethnic population and small sample size. Additionally, several potential confounders (e.g., molecular disease characteristics, baseline inflammatory biomarkers, cytokine profiles) were excluded from the analyses, as complete datasets for these indicators could not be fully retrieved under this retrospective design. Accordingly, prospective multicenter studies with larger cohorts and standardized clinical data collection protocols are needed to refine our kinetic model and conduct external validation in subsequent investigations. Second, given the use of an IL-6 knockdown CAR-T cell product and the limited number of severe CRS cases in this study, further validation is required to determine whether the exposure-toxicity cutoffs identified herein are applicable to other CD19-targeted CAR-T cell products.

## 5 Conclusion

In conclusion, we characterized the population cellular kinetics of CAR-T cells in 131 patients with r/r B-ALL using a population cellular kinetics model. The typical kinetics of rapid expansion and long-term persistence of CAR-T cells were elucidated. Additionally, CRS incidence during CAR-T cell therapy was found to be correlated with the exposure of CAR-T cells, and the optimal cutoff values for the early cellular kinetic parameters C_max_ and AUC_0-28day_ were exploratively identified to evaluate the occurrence of severe CRS.

## Data Availability

The raw data supporting the conclusions of this article will be made available by the authors, without undue reservation.

## Supplementary material

SUPPLEMENTARY FIGURE S1 Exploratory analysis of continuous covariates and categorical covariates plotted against post hoc random-effects estimates (continuation of Figure 1).
