# Supplementary material for: Population cellular kinetics and exposure-CSR analysis of CAR-T cells in Chinese patients with relapsed/refractory B-ALL
Source: Front Pharmacol. 2026 Oct 9;17:1878984. doi: 10.3389/fphar.2026.1878984 (PMC13690635; doi:10.3389/fphar.2026.1878984)
Supplement: Supplementary file 1 [file Image1.pdf]

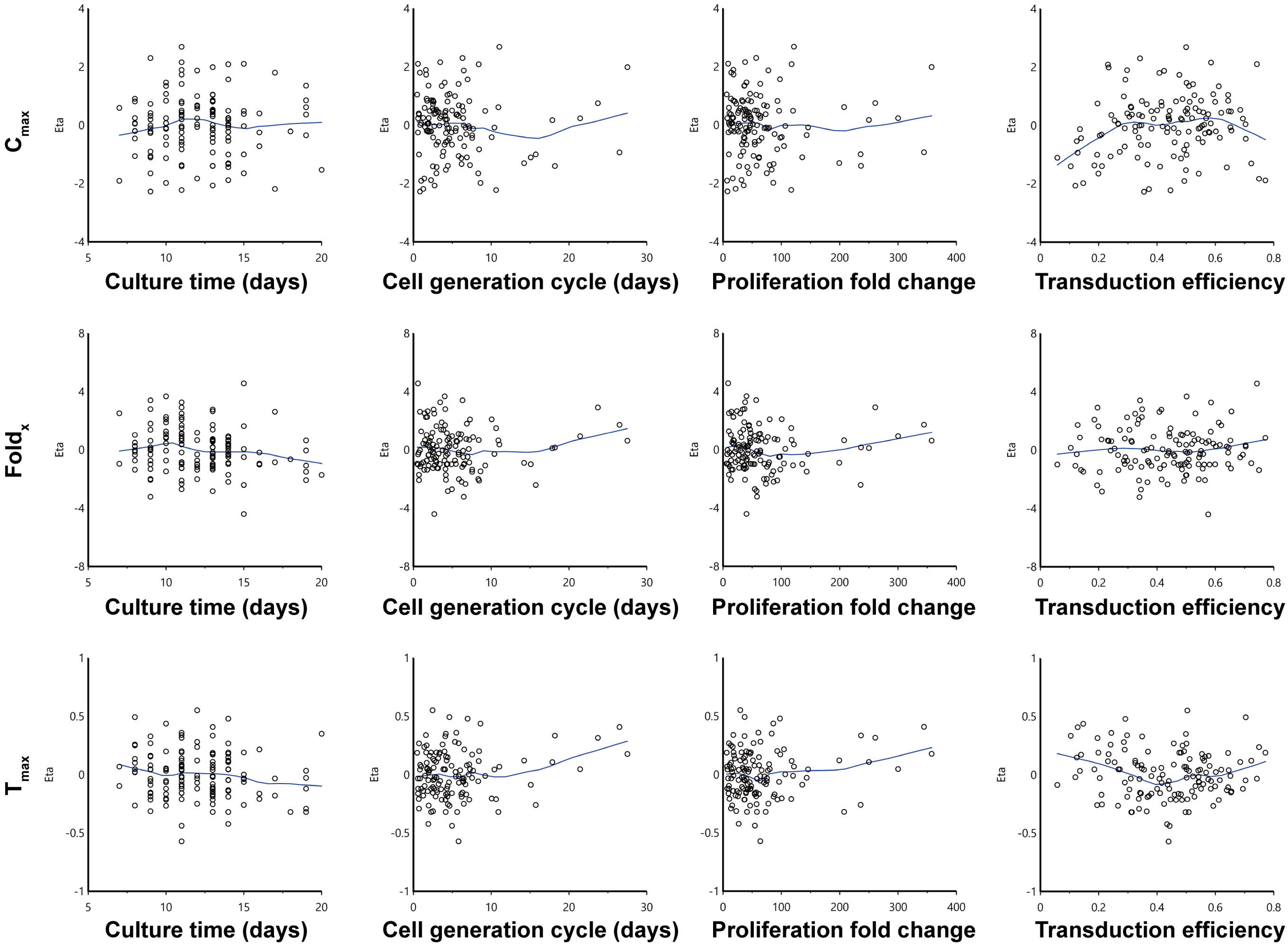
Cmax
Foldx
Tmax
Eta
Culture time (days)
Cell generation cycle (days)
Proliferation fold change
Transduction efficiency
